# Attitudes Toward Using COVID-19 mHealth Tools Among Adults With Chronic Health Conditions: Secondary Data Analysis of the COVID-19 Impact Survey

**DOI:** 10.2196/24693

**Published:** 2020-12-17

**Authors:** Marlene Camacho-Rivera, Jessica Yasmine Islam, Argelis Rivera, Denise Christina Vidot

**Affiliations:** 1 Department of Community Health Sciences State University of New York Downstate Health Sciences University Brooklyn, NY United States; 2 University of North Carolina Lineberger Comprehensive Cancer Center Chapel Hill, NC United States; 3 Department of Medicine Icahn School of Medicine at Mount Sinai New York, NY United States; 4 University of Miami School of Nursing and Health Studies Coral Gables, FL United States

**Keywords:** smartphone, mHealth, COVID-19, chronic health conditions, health disparities, chronic disease, attitude, perception, data analysis, contact tracing, mobile app, disparity

## Abstract

**Background:**

Adults with chronic conditions are disproportionately burdened by COVID-19 morbidity and mortality. Although COVID-19 mobile health (mHealth) apps have emerged, research on attitudes toward using COVID-19 mHealth tools among those with chronic conditions is scarce.

**Objective:**

This study aimed to examine attitudes toward COVID-19, identify determinants of COVID-19 mHealth tool use across demographic and health-related characteristics, and evaluate associations between chronic health conditions and attitudes toward using COVID-19 mHealth tools (eg, mHealth or web-based methods for tracking COVID-19 exposures, symptoms, and recommendations).

**Methods:**

We used nationally representative data from the COVID-19 Impact Survey collected from April to June 2020 (n=10,760). Primary exposure was a history of chronic conditions, which were defined as self-reported diagnoses of cardiometabolic, respiratory, immune-related, and mental health conditions and overweight/obesity. Primary outcomes were attitudes toward COVID-19 mHealth tools, including the likelihood of using (1) a mobile phone app to track COVID-19 symptoms and receive recommendations; (2) a website to track COVID-19 symptoms, track location, and receive recommendations; and (3) an app using location data to track potential COVID-19 exposure. Outcome response options for COVID-19 mHealth tool use were extremely/very likely, moderately likely, or not too likely/not likely at all. Multinomial logistic regression was used to compare the likelihood of COVID-19 mHealth tool use between people with different chronic health conditions, with not too likely/not likely at all responses used as the reference category for each outcome. We evaluated the determinants of each COVID-19 mHealth intervention using Poisson regression.

**Results:**

Of the 10,760 respondents, 21.8% of respondents were extremely/very likely to use a mobile phone app or a website to track their COVID-19 symptoms and receive recommendations. Additionally, 24.1% of respondents were extremely/very likely to use a mobile phone app to track their location and receive push notifications about whether they have been exposed to COVID-19. After adjusting for age, race/ethnicity, sex, socioeconomic status, and residence, adults with mental health conditions were the most likely to report being extremely/very or moderately likely to use each mHealth intervention compared to those without such conditions. Adults with respiratory-related chronic diseases were extremely/very (conditional odds ratio 1.16, 95% CI 1.00-1.35) and moderately likely (conditional odds ratio 1.23, 95% CI 1.04-1.45) to use a mobile phone app to track their location and receive push notifications about whether they have been exposed to COVID-19.

**Conclusions:**

Our study demonstrates that attitudes toward using COVID-19 mHealth tools vary widely across modalities (eg, web-based method vs app) and chronic health conditions. These findings may inform the adoption of long-term engagement with COVID-19 apps, which is crucial for determining their potential in reducing disparities in COVID-19 morbidity and mortality among individuals with chronic health conditions.

## Introduction

Since the declaration of the COVID-19 pandemic in the United States, over 7 million COVID-19 cases have been identified, and more than 200,000 lives have been lost [1]. Public health strategies for reducing the transmission of COVID-19 have included the enactment of policies, such as quarantine and social distancing, closures of nonessential businesses, and recommendations for preventive behaviors, such as hand washing and wearing face masks [2,3]. Emerging studies have reported that the implementation of policies and adherence to preventive best practices have varied widely in different geographic locations and demographic subgroups [4,5]. Public health and clinical researchers have reported on the disproportionate burden of COVID-19 morbidity and mortality among racial and ethnic minorities, older adults, and individuals with preexisting chronic health conditions [6-21]. Based on clinical and population-based studies, non-Hispanic Black and Latino individuals and communities are at increased risk for COVID-19 exposure [17,19,20,22], morbidity [8,9,23,24], and mortality [7,25,26]. Numerous chronic health conditions, including hypertension [8,23,26-29], diabetes [11,23,27,28,30], cancer [15,31-33], chronic obstructive pulmonary disease (COPD) [34-36] and asthma [28,37-39], and obesity [40-43] have been associated with an increased risk for poor COVID-19 outcomes.

The proliferation of COVID-19 mobile health (mHealth) tools for tracking COVID-19 statistics, monitoring potential COVID-19 symptoms, and reducing the social and mental health impacts of the COVID-19 pandemic has emerged simultaneously with the emergence of the pandemic [44-64]. Several COVID-19 apps that have been developed for public health surveillance provide up-to-date statistics, including the number of new cases, hospitalizations, and confirmed deaths [46,62,65]. Among the apps that allow for real-time symptom monitoring, trackers and telemedicine systems have helped elucidate the frequency of symptoms associated with COVID-19, thereby allowing patients and health care providers the opportunity to respond early to symptom progression [44,47,48,66]. Reviews of contact tracing apps have highlighted their effectiveness in improving the spatiotemporal reporting of new cases, management and follow-up of COVID-19 cases, and education on preventive behaviors [52,53,57,58,67-70]. To address the mental and social impacts of the COVID-19 pandemic, several apps have focused on reducing social isolation, providing positive coping strategies, and monitoring mental health symptoms [55,56,63,71-74]. Although researchers have noted the utility of COVID-19 apps in providing education and public health surveillance amid a rapidly evolving landscape, reviews of COVID-19 apps have highlighted several barriers to the availability, safety, and long-term sustainability of these technologies, including cost, the use of evidence-based guidelines, and user-centered design considerations for functionality and content [45,61,75-77]. Although differences in user preferences and perceptions of COVID-19 susceptibility based on individual risk factors exist across various social and demographic groups, few studies have examined attitudes toward COVID-19 mHealth tools among adults with and without preexisting health conditions.

The aims of this study were (1) to identify differences in attitudes toward COVID-19 mHealth tools across individuals with chronic health conditions; (2) to evaluate associations between having a preexisting chronic health condition and attitudes toward using COVID-19 mobile-based apps and websites to track potential COVID-19 exposure and symptoms; and (3) to identify determinants of mHealth intervention use. To accomplish these study objectives, we used publicly available data from the COVID-19 Impact Survey, which was designed to provide a nationally representative sample of the US adult population and offer national insights about the American population’s experiences during the COVID-19 pandemic.

## Methods

### COVID-19 Impact Survey Dataset

Data for our analyses were obtained from the publicly available COVID-19 Household Impact Survey, which was conducted by the nonpartisan and objective research organization NORC (National Opinion Research Center) at the University of Chicago for the Data Foundation. The COVID-19 Household Impact Survey is a philanthropic effort to provide national and regional statistics about physical health, mental health, economic security, and social dynamics in the United States [78]. The survey is designed to provide weekly estimates of the US adult (ie, aged ≥18 years) household population nationwide. Currently, data from week 1 (ie, April 20-26, 2020), week 2 (ie, May 4-10, 2020), and week 3 (ie, May 30 to June 8, 2020) are available. These data were merged for this analysis. As the AmeriSpeak analytic sample of the COVID-19 Impact Survey was derived from deidentified publicly available data, institutional review board approval was not required for this study.

AmeriSpeak is a probability-based panel that is funded and operated by the NORC at the University of Chicago. It is designed to be representative of the US household population. During the initial recruitment phase of the AmeriSpeak panel for the COVID-19 Impact Survey, randomly selected US households were sampled using area probability and address-based sampling methods. These sampled households were then contacted by US mail, telephone, and field interviewers (ie, face-to-face interview). The panel provides sample coverage for approximately 97% of the US household population. Those excluded from the sample include people with post office box–only addresses, addresses not listed in the US Postal Service Delivery Sequence File, and newly constructed dwellings. Although most AmeriSpeak households can participate in surveys via the internet, households without web access are able to participate in AmeriSpeak surveys by telephone. Interviews were conducted in English and Spanish. Interviews were conducted with adults who were aged ≥18 years and represented the 50 states and the District of Columbia. Panelists were offered a US $5 monetary incentive for completing the survey. With regard to the number of participants invited and percentage of interviews completed by week, 11,133 panelists were invited and 19.7% of interviews were completed in week 1, 8570 panelists were invited and 26.1% of interviews were completed in week 2; and 10,373 panelists were invited and 19.7% of interviews were completed in week 3 [78]. The analytic sample includes 10,760 nationwide adults and is weighted to reflect the US population of adults aged ≥18 years. The demographic weighting variables were obtained from the 2020 Current Population Survey [79].

### Attitudes Toward Using COVID-19 mHealth Tools

Our primary outcomes for this analysis were participants’ attitudes and willingness toward using COVID-19 mHealth tools to track potential COVID-19 exposure and symptoms. To characterize attitudes toward the use of COVID-19 mHealth tools for tracking potential COVID-19 exposure, we used participant’s responses to the following question: “If this option was available to you, how likely would you install an app on your phone that tracks your location and sends push notifications if you might have been exposed to COVID-19”?

To characterize attitudes toward the use of COVID-19 mHealth tools for tracking potential COVID-19 symptoms and recommendations, we used participant’s responses to the following 2 questions: (1) “If this option was available to you, how likely would you use a website to log your symptoms and location and get recommendations about COVID-19”; and (2) “If this option was available to you, how likely would you install an app on your phone that asks you questions about your own symptoms and provides recommendations about COVID-19”?

For each of the 3 outcomes, the provided response options were extremely likely, very likely, moderately likely, not too likely, and not likely at all. Due to the small sample sizes, the extremely likely and very likely response options were combined into 1 category, and the not too likely and not likely at all response options were also combined into 1 category. This was done for each outcome.

### History of Chronic Health Conditions

The primary predictor for this analysis was participants’ self-reports on a chronic health condition. Within the survey, participants were asked to reply “yes, no, or not sure” to the following question: “Has a doctor or other health care provider ever told you that you have any of the following: Diabetes; High blood pressure or hypertension; Heart disease, heart attack or stroke; Asthma; Chronic lung disease or COPD; Bronchitis or emphysema; Allergies; a Mental health condition; Cystic fibrosis; Liver disease or end-stage liver disease; Cancer; a Compromised immune system; or Overweight or obesity.” Given the small sample sizes reported among certain categories of health conditions, we further aggregated health conditions into the following 5 categories: cardiometabolic (ie, diabetes, high blood pressure, heart disease, heart attack or stroke, and liver disease or end-stage liver disease), overweight/obesity, respiratory (ie, allergies, asthma, chronic lung disease or COPD, and bronchitis or emphysema), immune-related (ie, cystic fibrosis, cancer, and a compromised immune system), and mental health conditions.

### Covariates

The following covariates were included in the multivariable analyses: age categories (ie, 18-29, 30-44, 45-59, ≥60 years), sex (ie, male or female), race/ethnicity categories (ie, non-Hispanic White, non-Hispanic Black, Hispanic or Latino, Asian, and non-Hispanic other), education categories (ie, no high school diploma, high school graduate, some college, and baccalaureate or above), residence (ie, rural or urban), and household income (ie, <US $50,000, US $50,000-US $100,000, ≥US $100,000). These covariates were chosen based on the review of mHealth disparities literature and COVID-19 disparities literature [5,80-83].

### Data Analysis

Descriptive statistics are presented as percentages for all respondents unless otherwise labeled, and include a margin of error of 3.0 percentage points for 95% confidence intervals among all adults. Chi-square tests were used for the univariate comparison of categorical variables, including age, sex, race/ethnicity, education, income, residence, and chronic health conditions. For COVID-19 mHealth outcomes, we used multinomial logistic regression to compare the likelihood of COVID-19 mHealth tool use across people with different chronic health conditions after adjusting for age, sex, race/ethnicity, education, income, and residence. For each COVID-19 mHealth outcome, we compared participants’ likelihood of using COVID-19 mHealth tools to those in the not likely to use group (ie, the reference category). To estimate determinants of being extremely/very likely to use each COVID-19 mHealth intervention, we computed prevalence ratios via Poisson regression using the robust estimation of standard errors [84-86]. Potential variables for inclusion in the model were assessed using available sociodemographic variables and bivariate Poisson regression analysis. Due to the exploratory nature of this analysis, which used a predictive framework, an arbitrary *P* value of <.10 was used as criteria to include variables in the multivariable Poisson regression model. For multivariable Poisson regression models, adjusted prevalence ratios and 95% confidence intervals for each independent variable were calculated.

The Type I error was maintained at 5%. Due to the exploratory nature of this analysis, we did not include an adjustment for multiple comparisons [87,88]. All statistical analyses were conducted using Stata IC 15 (StataCorp LLC). Sampling weights were applied to provide results that were nationally representative of the US adult population. As such, absolute n values could not be reported.

## Results

### Descriptive COVID-19 Impact Survey Results

Tables 1 and 2 display the descriptive characteristics of the analytic sample. Of the 10,760 respondents, 61.6% of respondents were non-Hispanic White, 11.9% were non-Hispanic Black, 16.5% were Hispanic, and 8.6% were of another non-Hispanic race or ethnicity. With regard to education, 9.8% of respondents had less than a high school diploma, 28.2% had a high school diploma, 27.7% had some college education, and 34.3% had a baccalaureate degree or higher. With regard to age, 20.5% of participants were aged 18-29 years, 25.4% were aged 30-44 years, 24.3% were aged 45-59 years, and 29.8% were aged ≥60 years. With regard to sex, 48.3% of respondents were male and 51.7% were female. With regard to residence, 72.2% of participants lived in urban areas and 27.9% lived in rural or suburban areas.

Histories of chronic health conditions ranged from 15.5% of respondents reporting a history of mental health conditions to 37.8% reporting a history of cardiometabolic diseases. The most common chronic conditions reported were cardiometabolic diseases (37.8%) and overweight/obesity (33.1%). Furthermore, 21.8% of adults reported that they were extremely or very likely to install a mobile phone app to record symptoms and obtain recommendations about COVID-19, and 21.1% reported that they were extremely or very likely to use a website to log their symptoms and location and receive recommendations about COVID-19. Additionally, 24.1% of adults reported that they were extremely or very likely to install an app on their phone to track their location and receive push notifications about whether they may have been exposed to COVID-19.

**Table 1:** Demographic characteristics of the COVID-19 Impact Survey respondents (N=10,760) from April to June 2020. This survey is a nationally representative survey of the United States.

Characteristic		Total, %^a^ (95% CI)
**Age (years)**		
	18-29	20.5 (19.3-21.8)
	30-44	25.4 (24.4-26.5)
	45-59	24.3 (23.2-25.4)
	≥60	29.8 (28.6-30.9)
**Race/ethnicity**		
	Non-Hispanic White	61.6 (60.3-62.9)
	Non-Hispanic Black	11.9 (11.0-12.7)
	Hispanic	16.5 (15.5-17.7)
	Non-Hispanic Asian	5.1 (4.4-5.8)
	Other non-Hispanic race/ethnicity	3.5 (3.1-3.9)
**Sex**		
	Male	48.3 (47.0-49.6)
	Female	51.7 (50.4-53.0)
Employed in the past 7 days		49.7 (48.4-51.1)
**Education**		
	No high school diploma	9.8 (8.8-10.8)
	High school graduate	28.2 (27.0-29.6)
	Some college	27.7 (26.7-28.7)
	Baccalaureate or above	34.3 (33.1-35.5)
**Household income (US $)**		
	<50,000	45.8 (44.5-47.1)
	50,000-100,000	32.1 (30.9-33.3)
	≥100,000	22.1 (21.1-23.2)
**Residence**		
	Rural	9.1 (8.4-9.8)
	Suburban	18.8 (17.8-19.7)
	Urban	72.2 (71.0-73.3)
**Insurance type or health coverage plans**		
	Purchased plan	17.4 (16.4-18.5)
	Employer sponsored	51.7 (50.3-53.0)
	TRICARE	4.9 (4.4-5.4)
	Medicaid	23.5 (22.4-24.7)
	Medicare	25.3 (24.2-26.4)
	Dually eligible (ie, Medicare and Medicaid)	9.7 (9.0-10.4)
	VA	4.5 (4.0-5.0)
	Indian Health Service	1.2 (0.9-1.6)
	No insurance	8.8 (8.1-9.6)
Cardiometabolic-related chronic diseases^b^		37.8 (36.5-39.0)
Respiratory-related chronic diseases^c^		23.6 (22.5-24.7)
Immune-related chronic diseases^d^		13.1 (12.3-13.9)
Overweight/obesity		33.1 (31.9-34.3)
Mental health–related conditions^e^		15.5 (14.6-16.4)

^a^Absolute n values are not displayed due to the inclusion of survey weights to provide nationally representative estimates.

^b^Cardiometabolic-related chronic diseases include diabetes, high blood pressure, heart disease, and liver disease/end-stage liver disease.

^c^Respiratory-related diseases include asthma, chronic lung disease/chronic obstructive pulmonary disease, and bronchitis/emphysema.

^d^Immune-related chronic diseases include cystic fibrosis, cancer, and a compromised immune system.

^e^Mental health–related conditions include at least 1 mental health condition.

**Table 2:** Survey characteristics of the COVID-19 Impact Survey respondents (N=10,760) from April to June 2020. This survey is a nationally representative survey of the United States.

Questions and responses		Total, %^a^ (95% CI)
**How likely would be to participate in installing an app on your phone that asks you questions about your own symptoms and provides recommendations about COVID-19?**		
	Extremely/very likely	21.8 (20.7-22.9)
	Moderately likely	20.9 (19.8-22.1)
	Not too likely/not likely at all	57.3 (55.9-58.6)
**How likely would you be to participate in using a website to log your symptoms and location and get recommendations about COVID-19?**		
	Extremely/very likely	21.1 (20.1-22.2)
	Moderately likely	23.5 (22.3-24.7)
	Not likely	55.4 (54.1-56.7)
**How likely would you be to participate in installing an app on your phone that tracks your location and sends push notifications if you might have been exposed to COVID-19?**		
	Extremely/very likely	24.1 (22.9-25.2)
	Moderately likely	19.9 (18.8, 20.9)
	Not likely	56.0 (54.7-57.4)

^a^Absolute n values are not displayed due to the inclusion of survey weights to provide nationally representative estimates.

### Attitudes Toward Using COVID-19 mHealth Tools Across People With Chronic Health Conditions

As shown in Tables 3 and 4, differences in attitudes toward the use of COVID-19 mHealth tools emerged across various chronic health conditions. Compared to adults without mental health conditions, adults with a history of mental health conditions were significantly more likely to potentially download and use an app to track COVID-19 symptoms and recommendations (*P*=.004), potentially download and use an app to track their location and potential COVID-19 exposure (*P*<.001), and use a website to log COVID-19 symptoms and receive recommendations (*P*=.005). Compared to nonobese participants, respondents who reported being obese were significantly more likely to potentially download and use an app to track their location and potential COVID-19 exposure (*P*=.039). In the univariate analysis, no other significant differences in attitudes toward using COVID-19 mHealth tools were observed in respondents with a history of respiratory conditions or immune-related conditions.

**Table 3:** Attitudes toward mHealth interventions for COVID-19 testing and tracking to help slow the spread of the virus, stratified by cardiometabolic-related^a^, respiratory-related^b^, and immune-related^c^ chronic diseases.

Questions and responses			Cardiometabolic-related chronic disease				*P* value^d^		Respiratory-related chronic disease			*P* value^d^		Immune-related chronic disease				*P* value^d^		
			No		Yes				No		Yes				No		Yes			
			Total, %^e^ (95% CI)		Total, % (95% CI)				Total, % (95% CI)		Total, % (95% CI)				Total, % (95% CI)		Total, % (95% CI)			
**How likely would be to participate in installing an app on your phone that asks you questions about your own symptoms and provides recommendations about COVID-19?**							.02						>.99							.74
	Extremely/very	20.7 (19.40-22.2)		23.5 (21.7-25.3)				21.8 (20.6-23.1)		21.7 (19.5-24.1)				21.7 (20.5-22.9)		22.7 (19.8-25.8)				
	Moderately	21.8 (20.3-23.3)		19.5 (17.9-21.2)				20.9 (19.6-22.2)		21.0 (18.7-23.4)				20.9 (19.7-22.1)		21.3 (18.7-24.2)				
	Not likely	57.5 (55.7-59.2)		57.0 (54.9-59.1)				57.3 (55.8-58.8)		57.3 (54.5-60.0)				57.5 (56.0-58.9)		56.0 (52.6-59.4)				
**How likely would you be to participate in installing an app on your phone that tracks your location and sends push notifications if you might have been exposed to COVID-19?**							.65						.08							.55
	Extremely/very	23.9 (22.5-25.4)		24.4 (22.7-26.3)				23.8 (22.5-25.1)		25.1 (22.8-27.5)				23.9 (22.7-25.2)		25.2 (22.3-28.3)				
	Moderately	20.2 (18.9-21.7)		19.3 (17.7-20.9)				19.4 (18.2-20.6)		21.5 (19.3-24.0)				20.1 (18.9-21.2)		18.6 (16.1-21.5)				
	Not likely	55.9 (54.2-57.6)		56.3 (54.2-58.3)				56.9 (55.4-58.3)		53.4 (50.6-56.1)				56.0 (54.6-57.4)		56.1 (52.7-59.5)				
**How likely would you be to participate in using a website to log your symptoms and location and get recommendations about COVID-19?**							.45						.32							.91
	Extremely/very	20.6 (19.3-22.0)		22.0 (20.3-23.80				21.6 (20.4-22.9)		19.6 (17.6-21.8)				21.2 (20.0-22.4)		20.9 (18.2-23.80				
	Moderately	23.5 (22.0-25.0)		23.5 (21.8-25.3)				23.3 (22.0-24.7)		24.1 (21.8-26.6)				23.4 (22.2-24.7)		24.1 (21.2-27.2)				
	Not likely	55.9 (54.2-57.6)		54.5 (52.4-56.6)				55.1 (53.6-56.6)		56.2 (53.5-58.9)				55.4 (54.0-56.9)		55.0 (51.6-58.4)				

^a^Cardiometabolic-related chronic diseases include diabetes, high blood pressure, heart disease, and liver disease/end-stage liver disease.

^b^Respiratory-related chronic diseases include asthma, chronic lung disease/chronic obstructive pulmonary disease, and bronchitis/emphysema.

^c^Immune-related chronic diseases include cystic fibrosis, cancer, and a compromised immune system.

^d^Refers to Chi-square *P* values.

^e^Absolute n values are not displayed due to the inclusion of survey weights to provide nationally representative estimates.

**Table 4:** Attitudes toward mHealth interventions for COVID-19 testing and tracking to help slow the spread of the virus, stratified by mental health–related^a^ chronic diseases and overweight/obese.

Questions and responses		Mental health-related chronic disease		*P* value^b^	Overweight/obese		*P* value^b^
		No	Yes		No	Yes	
		Total, %^c^ (95% CI)	Total, % (95% CI)		Total, % (95% CI)	Total, % (95% CI)	
**How likely would be to participate in installing an app on your phone that asks you questions about your own symptoms and provides recommendations about COVID-19?**				<.001			.54
	Extremely/very	21.6 (20.4-22.8)	22.8 (20.2-25.7)		21.6 (20.2-23.0)	22.2 (20.4-24.1)	
	Moderately	20.2 (19.0-21.5)	24.7 (21.8-27.7)		21.3 (19.9-22.8)	20.1 (18.3-21.9)	
	Not likely	58.6 (56.7-59.6)	52.5 (49.2-55.8)		57.1 (55.4-58.7)	57.7 (55.5-59.9)	
**How likely would you be to participate in installing an app on your phone that tracks your location and sends push notifications if you might have been exposed to COVID-19?**				<.001			.04
	Extremely/very	23.6 (22.4-24.9)	26.7 (23.9-29.7)		23.1 (21.7-24.5)	26.1 (24.2-28.1)	
	Moderately	19.2 (18.1-20.4)	23.4 (20.6-26.5)		19.9 (18.7-21.3)	19.7 (18.0-21.6)	
	Not likely	57.2 (55.7-58.6)	49.9 (46.6-53.2)		57.0 (55.3-58.6)	54.2 (52.0-56.3)	
**How likely would you be to participate in using a website to log your symptoms and location and get recommendations about COVID-19?**				.01			.26
	Extremely/very	20.9 (19.7-22.1)	22.5 (19.9-25.4)		21.2 (19.9-22.6)	21.0 (19.3-22.8)	
	Moderately	22.9 (21.6-24.1)	26.9 (24.1-30.0)		22.8 (21.4-24.3)	24.8 (22.9-26.8)	
	Not likely	56.3 (54.8-57.7)	50.5 (47.2-53.8)		55.9 (54.3-57.6)	54.2 (52.0-56.4)	

^a^Mental health–related chronic disease include at least 1 mental health condition.

^b^Refers to Chi-square *P* values.

^c^Absolute n values are not displayed due to the inclusion of survey weights to provide nationally representative estimates.

### Multinomial Logistic Regression Results for Attitudes Toward Using COVID-19 mHealth Tools Across People With Chronic Health Conditions

**Figure 1:**
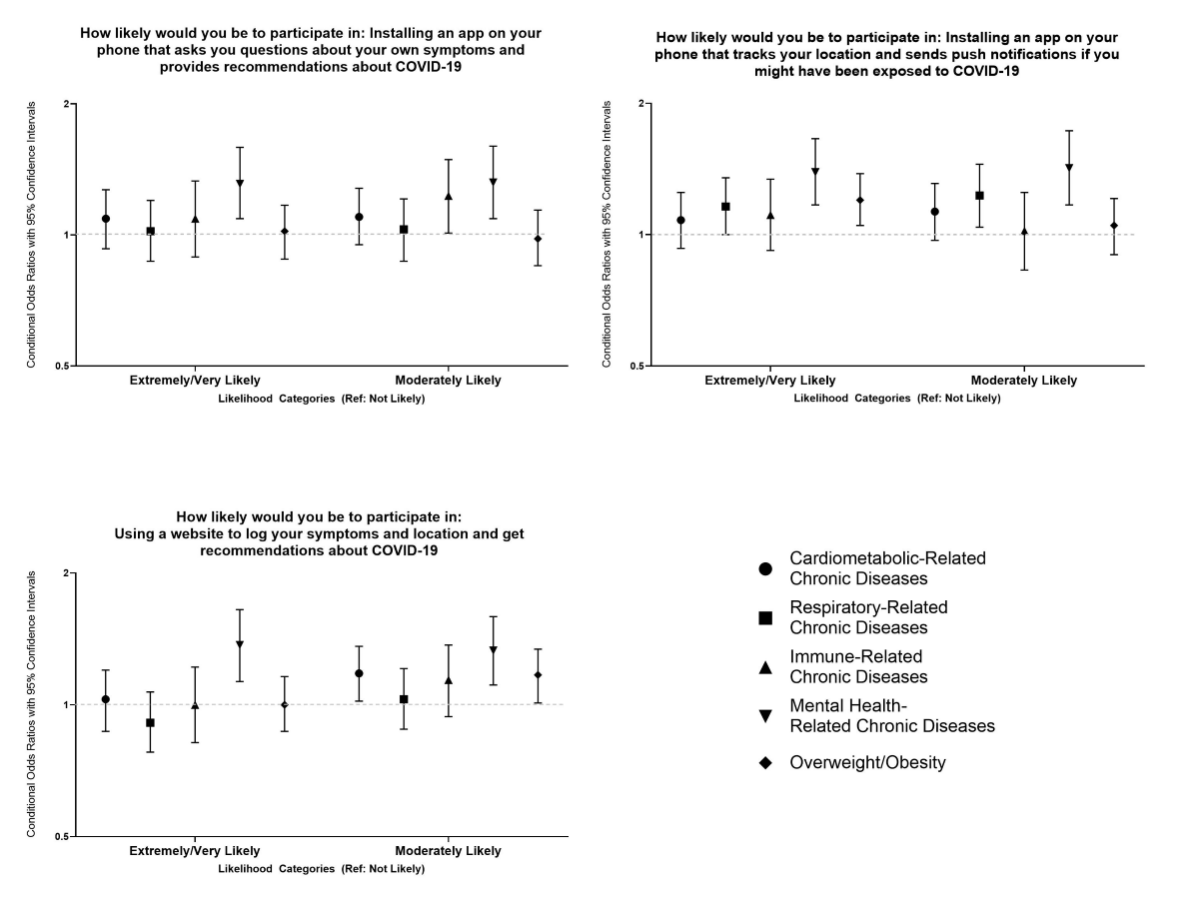
Associations between COVID-19 mHealth intervention acceptability and different chronic disease groups.

Figure 1 displays the multivariable results for the multinomial logistic regression models for each COVID-19 mHealth outcome and chronic health condition category. These models were adjusted for age, sex, race/ethnicity, education, income, and residence. The point estimates are available in Table S1 in Multimedia Appendix 1. Compared to adults without a history of cardiometabolic diseases, adults with cardiometabolic conditions were moderately likely to use a website to log their COVID-19 symptoms (conditional odds ratio [cOR] 1.18, 95% CI 1.02-1.36). Compared to adults without a history of respiratory conditions, adults with a history of respiratory diseases were moderately likely to download and use an app to track their location and potential COVID-19 exposure (cOR 1.23, 95% CI 1.04-1.45). Compared to adults without immune-related conditions, adults with a history of immune-related diseases were moderately likely to potentially download and use an app to track COVID-19 symptoms and receive health recommendations about COVID-19 (cOR 1.23, 95% CI 1.01-1.49).

Compared to adults without a history of mental health conditions, adults a history of mental health conditions were extremely likely and moderately likely to potentially download and use an app to track COVID-19 symptoms and receive health recommendations about COVID-19 (cOR 1.31, 95% CI 1.09-1.59; cOR 1.32, 95% CI 1.09-1.60, respectively). Adults with a history of mental health conditions were extremely likely and moderately likely to download and use an app to track location and potential COVID-19 exposure compared to adults without a history of mental health conditions (cOR 1.39, 95% CI 1.17-1.66; cOR 1.42, 95% CI 1.17-1.73, respectively). Compared to adults without a history of mental health conditions, adults with a history of mental health conditions were extremely likely and moderately likely to use of a website to log their COVID-19 symptoms (cOR 1.37, 95% CI 1.13-1.65; cOR 1.33, 95% CI 1.11-1.59, respectively).

Compared to nonobese participants, adults who reported being obese were extremely likely to download and use an app to track location and potential COVID-19 exposure (cOR 1.20, 95% CI 1.05-1.38). Participants who were obese were also moderately likely to use a website to log their COVID-19 symptoms compared to nonobese participants (cOR 1.17, 95% CI 1.01-1.34).

### Determinants of Being Extremely/Very Likely to Use COVID-19 mHealth Interventions Among US Adults

Table 5 summarizes the results of the analysis for identifying determinants of using each mHealth intervention among US adults. Across each mHealth intervention, there were several common determinants. Women had a higher prevalence of being extremely/very likely to use each mHealth intervention than men. Compared to those with a college degree, adults with a high school degree and some college education had a lower prevalence of being extremely/very likely to use each mHealth intervention. Racial/ethnic minorities, including non-Hispanic Black, Hispanic, and non-Hispanic Asian adults, had a higher prevalence of being extremely/very likely to use each mHealth intervention compared to non-Hispanic White adults. Additionally, adults with at least 1 COVID-19 related symptom had a higher prevalence of being extremely/very likely to install and use mHealth apps.

**Table 5:** Determinants of being extremely/very^a^ likely to use COVID-19 mHealth interventions based on data from the COVID-19 Impact Survey collected from April to June 2020. The survey is a nationally representative survey of the United States.

Variable		Installing an app on your phone that asks you questions about your own symptoms and provides recommendations about COVID-19, adjusted PR^b^ (95% CI)	Installing an app on your phone that tracks your location and sends push notifications if you might have been exposed to COVID-19, adjusted PR (95% CI)	Using a website to log your symptoms and location and get recommendations about COVID-19, adjusted PR (95% CI)
**Age (years)**				
	18-29	0.71 (0.59-0.86)	—^c,d^	0.74 (0.61-0.91)
	30-44	0.80 (0.70-0.92)	—	0.90 (0.78-1.03)
	45-59	1.03 (0.90-1.17)	—	1.02 (0.89-1.16)
	≥60	Ref^e^	—	Ref
**Sex**				
	Male	Ref	Ref	Ref
	Female	1.12 (1.01-1.24)	1.12 (1.02-1.23)	1.11 (1.00-1.23)
**Education**				
	No high school diploma	1.07 (0.87-1.33)	0.99 (0.80-1.21)	0.92 (0.73-1.16)
	High school graduate	0.75 (0.65-0.87)	0.74 (0.64-0.85)	0.68 (0.59-0.80)
	Some college	0.80 (0.71-0.90)	0.79 (0.71-0.88)	0.78 (0.70-0.88)
	Baccalaureate or above	Ref	Ref	Ref
**Race/ethnicity**				
	Non-Hispanic White	Ref	Ref	Ref
	Non-Hispanic Black	1.58 (1.36-1.83)	1.32 (1.14-1.53)	1.67 (1.44-1.93)
	Hispanic	1.49 (1.29-1.73)	1.29 (1.13-1.47)	1.47 (1.27-1.70)
	Non-Hispanic Asian	1.84 (1.47-2.30)	1.73 (1.43-2.10)	1.74 (1.39-2.17)
	Other non-Hispanic race/ethnicity	1.18 (0.90-1.55)	1.07 (0.83-1.38)	1.23 (0.94-1.61)
At least 1 COVID-19–related symptom^f^		1.13 (1.02-1.25)	1.19 (1.08-1.31)	—^d^
At least 1 chronic disease^g^		—^d^	—^d^	—^d^
**Region**				
	Northeast	Ref	—^d^	Ref
	Midwest	0.84 (0.71-0.99)	—	0.76 (0.64-0.90)
	South	0.97 (0.83-1.13)	—	0.85 (0.73-0.99)
	West	0.83 (0.71-0.98)	—	0.79 (0.67-0.92)
Employed		0.85 (0.76-0.95)	0.85 (0.77-0.94)	0.78 (0.70-0.88)
Uninsured		—^d^	—^d^	0.90 (0.74-1.10)
**Household income (US $)**				
	<50,000	0.96 (0.84-1.10)	0.82 (0.73-0.93)	0.86 (0.74-1.00)
	50,000-100,000	0.91 (0.80-1.04)	0.86 (0.76-0.97)	0.85 (0.74-0.98)
	≥100,000	Ref	Ref	Ref
**Residence**				
	Rural	0.95 (0.78-1.17)	1.04 (0.87-1.25)	0.98 (0.81-1.19)
	Suburban	0.98 (0.85-1.12)	0.95 (0.83-1.08)	0.93 (0.80-1.07)
	Urban	Ref	Ref	Ref

^a^Respondents were asked if they were extremely likely, very likely, moderately likely, not too likely, or not at all likely to participate in each mHealth intervention. We categorized those who responded with extremely/very likely to participate as exposed (ie, exposed=1) and those who gave other responses as unexposed (ie, unexposed=0).

^b^PR: prevalence ratio.

^c^Not available.

^d^The corresponding *P* value was >.10 for all categories of the variable in unadjusted analyses

^e^Ref: the referent group.

^f^COVID-19–related symptoms include fever, chills, runny or stuffy nose, chest congestion, skin rash, cough, sore throat, sneezing, muscle or body aches, headaches, fatigue or tiredness, shortness of breath, abdominal discomfort, nausea or vomiting, diarrhea, changed or lost sense of taste or smell, and loss of appetite.

^g^Chronic disease include diabetes, high blood pressure, heart disease/heart attack/stroke, asthma, chronic obstructive pulmonary disease, bronchitis or emphysema, cystic fibrosis, liver disease, cancer, a compromised immune system, and overweight/obesity.

## Discussion

### Principal Results

This study analyzed differences and associations between attitudes toward using COVID-19 mHealth tools and various chronic health conditions. Compared to adults without a history of chronic health conditions, adults with chronic health conditions were more likely to potentially use COVID-19 apps or websites to monitor potential COVID-19 exposure and symptoms. Yet, attitudes toward COVID-19 mHealth tools varied significantly across people with different chronic disease conditions and modalities (ie, app vs website). Our study findings highlight the potential for mHealth tools to improve disease self-management and reduce health disparities among individuals with chronic health conditions.

Our study highlighted disparities in attitudes toward COVID-19 mHealth tools across age, sex, race/ethnicity, education, and region. We observed that women in our sample were more likely to report positive attitudes toward using an app or website to track potential COVID-19 exposures or symptoms compared to men. This is consistent with prior studies that focused on disparities in mHealth use among the general population [80,89]. Previous studies have also documented lower rates of using mHealth tools among individuals with lower socioeconomic backgrounds compared to individuals with higher socioeconomic backgrounds [81,90]. In our study, irrespective of self-reported chronic health conditions, participants with lower levels of education were less likely to use an app or website for tracking COVID-19 symptoms or exposure compared to respondents with a college degree or higher. Similar patterns were observed in the different income groups [91].

Our study also highlighted several novel findings. The first was that respondents with a racial/ethnic minority background had a greater likelihood of using COVID-19 mHealth tools than non-Hispanic White respondents in our entire sample. Hispanic (adjusted prevalence ratio [aPR] 1.49, 95% CI 1.29-1.73), Asian (aPR 1.84, 95% CI 1.47-2.30), and non-Hispanic Black (aPR 1.58, 95% CI 1.36-1.83) respondents were significantly more likely to potentially use mHealth tools for tracking COVID-19 exposure and symptoms. These attitudes may indicate greater awareness among racial and ethnic minority communities with regard to increased susceptibility to COVID-19 morbidity and mortality [22,25,92]. Additionally, the use of mHealth tools has been increasing among all racial/ethnic groups over the last decade. This is indicative of all racial/ethnic groups having greater access to mHealth tools. This is also indicative of the potential that mHealth tools have for reducing racial/ethnic disparities in chronic disease management, morbidity, and mortality.

The second novel finding was observed when examining age-related disparities in COVID-19 mHealth attitudes. Contrary to many prior studies that have documented increased mHealth tool use among younger adults, respondents aged 18-29 years reported being less likely to use apps or websites for tracking COVID-19 symptoms and exposure compared to participants aged ≥60 years. These differences may indicate that older adults have a greater awareness of the risks of COVID-19 than younger adults. These differences may also indicate potential misperceptions of COVID-19 risk among younger populations [93,94].

We also observed geographic differences in attitudes toward COVID-19 mHealth tools with participants from the Midwest, South, and West, who reported less interest in using apps or websites to track COVID-19 symptoms and exposure compared to respondents from the Northeast. These disparities are consistent with prior studies that focused on regional differences in the digital divide, as well as studies of COVID-19 preventive behaviors across the United States [95]. Our findings may also reflect geographic differences in perceived susceptibility to COVID-19 at the time the survey was administered, given the disproportionate burden of COVID-19 cases in the Northeast during the onset of the pandemic [96,97].

Our findings are important, given the accumulating evidence for cardiovascular diseases, obesity, and diabetes being risk factors for COVID-19 incidence and mortality. In our previous study, which analyzed COVID-19 preventive behaviors among US adults, we observed that adults with chronic health conditions were significantly more likely to engage in many COVID-19 preventive behaviors, such as hand washing, using a face mask, and maintaining appropriate social distancing in public [5,33]. The use of mHealth tools for COVID-19 education, risk reduction, and symptom monitoring may complement existing prevention strategies [98]. Our findings also highlight potential opportunities for mHealth tools to reduce racial/ethnic and age disparities in COVID-19 exposure and outcomes [99,100].

Lastly, our findings highlight the potential that mHealth tools have for reducing chronic disease disparities in disease management and outcomes beyond the COVID-19 pandemic. Although there has been a proliferation of mHealth tools for people with chronic health conditions, the acceptability and long-term use of mHealth tools have varied considerably across people with different chronic conditions [101,102]. Variability in mHealth tool use across people with chronic health conditions may be related to the quality of both the content and functionality of mHealth tools, as well as user-related preferences [103-107].

### Study Strengths and Limitations

Our results should be interpreted within the context of several limitations. First, a history of chronic health conditions was based on self-reported data rather than medical records. Therefore, there may be potential for recall or misclassification bias for the chronic health conditions reported in this analysis. Additionally, while we acknowledge the increasing importance of considering multiple chronic conditions, given the complexity of examining multiple chronic conditions by both number and the combination of different chronic condition types, the examination of attitudes toward using COVID-19 mHealth tools among individuals with multiple chronic conditions was beyond the scope of this analysis. Second, attitudes toward potential COVID-19 mHealth tool use were based on self-reports rather than downloads or monitoring patterns of actual mHealth tool usage. As such, there may be potential for social desirability bias in terms of the reported COVID-19 mHealth attitudes, which may not correlate with actual COVID-19 mHealth tool use.

Despite these limitations, our study has notable strengths. First, the sampling methods used to obtain a nationally representative sample of the US adult population provided the opportunity for increased generalizability. Our findings may be helpful to clinicians and public health officials when guiding mHealth messaging for COVID-19 prevention, symptom monitoring, and health recommendations. Additionally, our study results are drawn from a population that is diverse in terms of social and demographic characteristics and health statuses. Since the COVID-19 pandemic has exacerbated health inequities across different social groups and groups with chronic health conditions, public health strategies that consider the utility of mHealth approaches across diverse contexts will have a vital role in reducing COVID-19 disparities.

### Conclusions

Our study extends previous research in this field by analyzing attitudes toward various COVID-19 mHealth tools across a nationally representative sample of US adults with and without chronic health conditions. Future directions for research may include the examination of COVID-19 mHealth tool use among individuals with multiple chronic conditions and associations between COVID-19 mHealth tool use and COVID-19 knowledge and preventive behaviors. Our study results have several implications concerning the development of COVID-19 mHealth tools. As the use of mHealth apps has been found to impact the improvement of health outcomes across a range of chronic health conditions, the development of COVID-19 apps should focus on user preferences and consider differences in COVID-19 susceptibility across people with different chronic disease conditions. Combining big data analytic approaches with qualitative data from individuals with chronic health conditions may yield additional insights in increasing the acceptability of and long-term engagement with COVID-19 apps. Lastly, incorporating opportunities for the real-time tracking of potential COVID-19 exposure and symptoms among existing health apps for people with chronic conditions (eg, mental health conditions, cardiometabolic conditions, and respiratory diseases) may provide additional opportunities to strengthen prevention and early detection efforts among populations that are vulnerable to COVID-19 morbidity and mortality.

## Appendix

Multimedia Appendix 1 Table S1. Conditional odds ratios for evaluating associations between attitudes toward using COVID-19 mHealth tools and chronic disease status; estimates for Figure 1.

## Abbreviations

aPR: adjusted prevalence ratio
COPD: chronic obstructive pulmonary disease
cOR: conditional odds ratio
mHealth: mobile health
NORC: National Opinion Research Center
