# Supplementary material for: Attitudes Toward Using COVID-19 mHealth Tools Among Adults With Chronic Health Conditions: Secondary Data Analysis of the COVID-19 Impact Survey
Source: JMIR Mhealth Uhealth. 2020 Dec 17;8(12):e24693. doi: 10.2196/24693 (PMC7748389; doi:10.2196/24693)
Supplement: Multimedia Appendix 1 [file mhealth_v8i12e24693_app1.docx]

|  |  |  |  |  |  |  |  |  |  |  |  |  |  |  |  |  |
| --- | --- | --- | --- | --- | --- | --- | --- | --- | --- | --- | --- | --- | --- | --- | --- | --- |
| **Table S1.** Conditional odds ratios to evaluate associations between attitudes toward using COVID-19 mHealth tools and chronic disease status; estimates for Figure 1. | | | | | | | | | | | | | | | | |
| There are some options for testing and tracking people who may have COVID-19 in order to help slow the spread of this virus. If these options were available to you, how likely would you be to participate in them? | | | | | | | | | | | | | | | |  |
|  |  |  |  |  |  |  |  |  |  |  |  |  |  |  |  |  |
|  | Cardiometabolic-Related | | | Respiratory-Related | | | Immune-Related | | | Mental Health-Related | | | Overweight/Obesity | | |  |
| Installing an app on your phone that asks you questions about your own symptoms and provides recommendations about COVID-19 |  |  |  |  |  |  |  |  |  |  |  |  |  |  |  |  |
| Extremely/Very Likely | 1.09 | 0.93 | 1.27 | 1.02 | 0.87 | 1.20 | 1.09 | 0.89 | 1.33 | 1.31 | 1.09 | 1.59 | 1.02 | 0.88 | 1.17 |  |
| Moderately Likely | 1.10 | 0.95 | 1.28 | 1.03 | 0.87 | 1.21 | 1.23 | 1.01 | 1.49 | 1.32 | 1.09 | 1.60 | 0.98 | 0.85 | 1.14 |  |
| Not likely | Ref. |  |  | Ref. |  |  | Ref. |  |  | Ref. |  |  | Ref. |  |  |  |
|  |  |  |  |  |  |  |  |  |  |  |  |  |  |  |  |  |
| Installing an app on your phone that tracks your location and sends push notifications if you might have been exposed to COVID-19 |  |  |  |  |  |  |  |  |  |  |  |  |  |  |  |  |
| Extremely/Very Likely | 1.08 | 0.93 | 1.25 | 1.16 | 1.00 | 1.35 | 1.11 | 0.92 | 1.34 | 1.39 | 1.17 | 1.66 | 1.20 | 1.05 | 1.38 |  |
| Moderately Likely | 1.13 | 0.97 | 1.31 | 1.23 | 1.04 | 1.45 | 1.02 | 0.83 | 1.25 | 1.42 | 1.17 | 1.73 | 1.05 | 0.90 | 1.21 |  |
| Not likely | Ref. |  |  | Ref. |  |  | Ref. |  |  | Ref. |  |  | Ref. |  |  |  |
|  |  |  |  |  |  |  |  |  |  |  |  |  |  |  |  |  |
| Using a website to log your symptoms and location and get recommendations about COVID-19 |  |  |  |  |  |  |  |  |  |  |  |  |  |  |  |  |
| Extremely/Very Likely | 1.03 | 0.87 | 1.20 | 0.91 | 0.78 | 1.07 | 1.00 | 0.82 | 1.22 | 1.37 | 1.13 | 1.65 | 1.00 | 0.87 | 1.16 |  |
| Moderately Likely | 1.18 | 1.02 | 1.36 | 1.03 | 0.88 | 1.21 | 1.14 | 0.94 | 1.37 | 1.33 | 1.11 | 1.59 | 1.17 | 1.01 | 1.34 |  |
| Not likely | Ref. |  |  | Ref. |  |  | Ref. |  |  | Ref. |  |  | Ref. |  |  |  |
| Models adjusted for: sex, age, race/ethnicity, educational level, insurance status, area of residence (urban/rural) | | | | | | | | | | | | | | | |  |
